# Radiotherapy for Soft Tissue Sarcoma of the Proximal Lower Extremity

**DOI:** 10.1155/2010/829498

**Published:** 2010-10-14

**Authors:** Brendan Prendergast, John B. Fiveash, C. Parker Gibbs, Mark T. Scarborough, Daniel J. Indelicato

**Affiliations:** ^1^Department of Radiation Oncology, University of Alabama at Birmingham, Birmingham, AL 35294-1150, USA; ^2^Department of Orthopedics, College of Medicine, University of Florida, Gainesville, FL 32610, USA; ^3^Department of Radiation Oncology, College of Medicine, University of Florida, Gainesville, FL 32610, USA; ^4^University of Florida Proton Therapy Institute, Jacksonville, FL 32206, USA

## Abstract

Soft-tissue sarcoma (STS) is a histopathologically diverse group of tumors accounting for approximately 10,000 new malignancies in the US each year. The proximal lower extremity is the most common site for STS, accounting for approximately one-third of all cases. Coordinated multimodality management in the form of surgery and radiation is often critical to local control, limb preservation, and functional outcome. Based on a review of currently available Medline literature and professional experience, this paper provides an overview of the treatment of STS of the lower extremity with a particular focus on the modern role of radiotherapy.

## 1. Introduction

Soft tissue sarcomas (STS) are a relatively rare, histopathologically diverse group of neoplasms arising from mesenchymal cells including adipose, muscle, and connective tissues. The natural history of STS typically involves growth and compression of surrounding structures, rather than direct invasion of surrounding tissues. The distant spread of sarcoma is characteristically via early hematogenous spread, most often to the lungs [1]. Lymphatic spread of sarcoma is rare but may occur with certain histologic subtypes [2]. The Centers for Disease Control (CDC) estimated that 10,660 new cases of STS would occur in 2009, with approximately 3,820 deaths as a result [3]. Estimates show a relatively even distribution of STS between male and female patients, with no apparent predilection for particular ethnicities or races [3]. Despite a low overall incidence, STS is a fairly common entity in radiation oncology clinics as level-one evidence from several randomized controlled trials supports a multidisciplinary approach [4–6].

Although STS can occur at any body site, the proximal lower extremity (or thigh) is the predominant site and, therefore, forms the basis of this paper [1, 7]. Despite the array of histological subtypes of STS, cases occurring in the lower extremity are most commonly liposarcoma, undifferentiated pleomorphic sarcoma, tenosynovial sarcoma, fibrosarcoma, and epitheliod sarcoma.

## 2. Discussion

### 2.1. Anatomy

Enneking described the anatomy of the thigh by separating it into 3 compartments—anterior, medial, and posterior—with both oncologic and functional significance [8]. These distinctions have been widely used to describe the anatomy of the thigh, especially as it relates to STS [9]. The anterior compartment is particularly important as it is the specific anatomic location of most proximal lower-extremity tumors [10]. Its content includes the femur, patella, quadriceps, sartorius, tensor fascia lata, and the critical neurovascular structures of the femoral canal. Including the sartorious is debatable because it is set apart in its own fascial envelope, some argue it is a compartment unto itself [11, 12]. The medial compartment contains the adductors, gracilis, pectineus, and neurovascular structures including the obturator artery and nerve and the profunda femoris vessels. The posterior compartment contains the semitendinosus, semimembranosus, biceps femoris, the posterior portion of the adductor magnus muscles, and the sciatic nerve [8].

### 2.2. Surgery

Surgical resection is a central component in the management of STS, but the importance is magnified when considering disease of the proximal lower extremity where most lesions are resectable. Historically, amputation was the procedure of choice as it yields excellent local control if adequate margins are obtained. Compartmental resection is an alternative procedure that offers good local control and limb preservation but has been associated with high morbidity and limited functional outcomes [13]. Current surgical approaches to lower-extremity STS focus on limb salvage and preserved functional status as a primary goal, with amputation reserved for cases where tumor bulk or presence of a vital surrounding structure prohibits satisfactory oncologic margins [14]. 

To reduce the morbidity associated with amputation, surgeons historically performed simple excisions of STS and observed local recurrence rates of 60% to 90% [15]. Early data on STS of the thigh from Enneking et al. showed that local recurrence rates were significantly lower when more radical procedures were employed (Table 1) [8]. Several studies looking at radical resection of STS at other extremity sites have confirmed Enneking's work, with reported local control rates ranging from 8% to 31%, suggesting that margin status is a prime influence on local control [15, 16]. In one study examining patients with tumors of the proximal lower extremity, Vraa and colleagues found that positive surgical margins were associated with a significantly higher rate of local recurrence, but not worse survival [17]. 

In fact, data suggest that surgical margin status may be the single most critical factor preventing local recurrence. In a retrospective analysis, Karakousis and colleagues found that local control with widely negative margins and surgery alone was better than surgery plus adjuvant radiation for margins less than 2 cm [18]. These findings suggest that surgery alone may be adequate, as long as margins are widely negative. Several other investigators have reported local-control rates approaching 90% in select patients treated by surgery alone [19–23]. However, these small retrospective studies do not use standardized patient selection criteria and are thus prone to bias. A recent prospective trial by Pisters and colleagues demonstrated that excellent local control and survival could be achieved with surgery alone in patients with T1-stage tumors of the extremities [24]. There is no surgical data focusing specifically on the proximal lower extremity, but Fabrizio et al. report local control and survival statistics comparable to those from the adjuvant radiotherapy data in a series of patients primarily with thigh tumors (44%) treated with surgery alone (Table 1) [25]. However, the Scandinavian data call into question this approach. In a retrospective review including 469 cases of STS of the thigh, adding radiotherapy to surgery improved local control irrespective of surgical margin, grade, or depth [26]. 

In summary, the data suggest that wide excision alone remains an option for well-selected patients with small superficial tumors. However, patients with STS of the thigh commonly present with large subfascial tumors or tumors abutting critical neurovascular structures, making this strategy less practical.

### 2.3. Combined Modality

Although surgery alone is feasible for carefully selected cases of STS of the proximal lower extremity, the standard of care for treatment of primary STS continues to be wide excision with radiotherapy before or after surgery. Early evidence supporting the use of radiotherapy in the treatment of STS came from retrospective data over 30 years ago [27, 28]. In the early 1980s, a small randomized controlled trial (RCT) conducted at the National Cancer Institute (NCI) compared amputation to limb-sparing surgery and demonstrated low rates of local recurrence following surgery plus radiotherapy and equivalent rates of disease-free survival and overall survival when compared to amputation [6]. This trial established limb preservation as a viable goal and the standard of care.

 However, it was not until the late 1990s when two larger RCTs comparing adjuvant radiotherapy to no radiotherapy have published that the benefit of radiotherapy in STS was firmly established [4, 5]. The timing (i.e., preoperative or postoperative) of radiotherapy and the modality of radiation delivery (i.e., brachytherapy or external-beam radiotherapy) remain areas of controversy (Table 1). Likewise, the use of adjuvant or neoadjuvant chemotherapy in nonmetastatic STS of the thigh is controversial and will be addressed later.

### 2.4. Preoperative Radiotherapy

Although postoperative radiotherapy was employed in the treatment of STS in the landmark RCTs, preoperative radiotherapy is an attractive option for a number of reasons. On a general radiobiological level, radiation is more effective in a well-vascularized oxygenated tissue bed. A more radiosensitive preoperative environment theoretically allows for lower doses and smaller field sizes and is thought to lead to better functional outcomes. These endpoints have been addressed in several different studies specifically on STS. Nielsen and colleagues demonstrated that preoperative radiotherapy for STS of the extremity yields smaller field sizes when compared to postoperative radiotherapy [29]. Likewise, preoperative radiotherapy safely and effectively allows a lower cumulative dose to the involved field and subsequently better functional outcomes when compared to postoperative radiotherapy [30–33]. Other reports indicate that preoperative radiotherapy may allow easier resection of the primary tumor because the peripheral fibrosis creates a palpable tumor capsule that is more amenable to resection without tumor violation [34].

Several analyses indicate that preoperative radiotherapy for STS yields similarly low rates of local recurrence and comparable survival statistics as postoperative irradiation [35–38]. Some studies indicate that preoperative irradiation yields better local control rates, especially with large tumors [39, 40]. However, preoperative radiation has been repeatedly associated with significant wound complications [35, 41–43]. Wound complications tend to be more common and more severe when the primary tumor is located in the thigh or elsewhere in the lower extremity [30, 34, 41, 44, 45]. One notable study from the Canadian National Cancer Institute comparing preoperative to postoperative radiation for STS is illustrative: acute wound complications in patients irradiated before surgery were nearly double than in the postoperative group [30]. In this study, complications were defined as secondary operations or procedures for wound repair with or without anesthesia, hospital admission for wound care, or persistent deep packing of wounds. A subset analysis demonstrates that wound-complication rates are higher in the proximal lower extremity, where 45% of patients treated preoperatively had wound complications. A large retrospective analysis focused on STS of the lower extremity from the MD Anderson Cancer Center (MDACC; Houston, TX) supports this trend. In this analysis, which included 263 (64%) lesions in the thigh, preoperative radiotherapy was associated with a 34% wound-complication rate compared with 16% in the postoperative cohort [46]. Lastly, data from the University of Florida, where neoadjuvant radiotherapy has been the treatment of choice for 30 years, provide interesting data relating to tumors of the proximal lower extremity. In a retrospective analysis of 209 patients (130 thigh primaries), the wound complication rate was 22%, with a significantly higher percentage of complications occurring in proximal lower extremity cases [34]. 

Wound complications are clearly important; they are inconvenient, uncomfortable and often lead to poor short-term functional outcomes [47, 48]. Additionally, wound complications may incur greater costs on the healthcare system by leading to further surgical intervention [46]. However, the subjective variable definition of a wound complication is problematic when reviewing these data.

### 2.5. Postoperative Radiotherapy

In the 1990s, two large randomized controlled trials established the utility of postoperative radiotherapy for STS of the extremity. Both studies found significant decreases in local recurrence, but no difference in other endpoints like overall survival or freedom from distant disease. Yang randomized 141 patients to receive adjuvant external-beam radiation or no radiation following limb-sparing surgery and reported separate results for patients with low- and high-grade tumors. Among the high grade group, approximately half of the patients had a tumor located in the proximal lower extremity. At median follow up of 9.3 years, local recurrence was 24% for patients receiving no radiation and zero percent for patients receiving adjuvant radiotherapy. Among the low-grade group, the majority of patients again had a tumor located in the proximal lower extremity. At median follow up of 9.9 years, local recurrence was 33% for patients receiving no radiation, compared with 0% for patients who received radiotherapy. These differences in local recurrence were statistically significant. Although tumors of the proximal lower extremity ultimately developed the most local recurrences, the authors did not evaluate whether location itself was statistically significant, presumably due to the low number of overall recurrences [4].

Pisters et al. randomized 164 patients to receive either brachytherapy or no brachytherapy following resection of STS of the extremity or trunk. After median follow up of 76 months, local control was significantly improved in the brachytherapy arm, while freedom from distant recurrence and overall survival remained equal between groups. The most specific anatomic descriptor is proximal extremity in 120 patients (73%), with no stratification between upper or lower extremity. On multivariate analysis, location of the tumor in a proximal extremity was not associated with a higher risk of local recurrence [5]. Like preoperative external-beam radiotherapy, postoperative brachytherapy has also been associated with high complication rates. Researchers from the same group reported that if brachytherapy catheters are loaded within 5 days postoperatively, the wound complication rates approached 50% [49]. However, with a modified technique of loading catheters later in the postoperative period, wound complication rates for the entire group dropped to less than 15% and were equal in both arms [49, 50].

Though postoperative external-beam radiation has a lower incidence of wound complications, data suggest that these patients experience a higher degree of morbidity from late radiation effects. This likely represents sequelae of larger field sizes and higher doses used in the devascularized postsurgical tissue. A large retrospective study from MD Anderson suggests that postoperative irradiation is associated with more long-term morbidity than preoperative radiotherapy [37]. Randomized prospective data from a Canadian NCI trial confirm this finding. In this trial, patients who received postoperative radiotherapy tended to have more fibrosis, edema, and joint stiffness; however, the results are not statistically significant [48]. These endpoints are important: patients who experience fibrosis, edema, or joint stiffness as a result of treatment have significantly greater disability and impairment as measured by several validated instruments [48]. However, the severity of disability resulting from radiation at any time in extremity sarcoma is debatable. Concurrent quality of life and performance evaluations in patients undergoing postoperative radiation have shown no difference from patients not treated with radiation [4]. It is important to note that although decreasing the dose or field size might be a worthwhile tactic to minimize the late effects of radiation, a review of 64 patients from the University of Chicago found that smaller field sizes significantly decreased local control [51]. Furthermore, evidence suggests that patients treated to a total dose less than 62.5 Gy have significantly poorer survival compared to patients treated to a higher dose [52]. Little data are available specifically addressing dose or field size in proximal lower-extremity tumors. 

One study is available detailing the experience of postoperative radiotherapy in the proximal lower extremity. Investigators at Memorial Sloan Kettering Cancer Center reviewed data for 255 patients treated with wide local excision and postoperative radiotherapy and found a 9% local recurrence rate. Local recurrence, distant metastasis-free survival, and overall survival did not differ significantly based on the anatomic compartment of tumor origin [10]. Interestingly, in this series of patients only with thigh primaries, tumor size and margin status were not associated with local recurrence, contrary to prior reports. 

Although less widespread, published data suggest that adjuvant brachytherapy is comparable to other techniques in terms of local control, distant metastasis-free survival, overall survival, and complications [5, 53]. However, recently presented retrospective data from Memorial Sloan-Kettering Cancer Center, where brachytherapy had been the standard of care, suggest that patients treated with external beam intensity-modulated radiation therapy (IMRT) had improved local control when compared to the brachytherapy cohort, despite having more negative prognostic indicators [54]. Further evaluation in the prospective setting would be useful.

### 2.6. Radiotherapy Alone

Although adjuvant radiotherapy has proven its efficacy, definitive treatment of proximal lower-extremity STS is rarely performed since nearly all tumors are resectable with modern limb-salvage procedures or amputation. However, in select cases, patients who may be medically inoperable or otherwise resistant to radical surgery have been treated by radiation alone. Use of this technique is limited; early reports of photon radiotherapy alone for STS yielded poor results. Notably, some of this subset of STS patients treated with radiation alone have had poor overall health and performance status. Such factors confound survival statistics more than local control statistics, and, therefore, overall survival is difficult to interpret. In the 1980s, Tepper and Suit reviewed 51 patients (including 14 thigh cases) with localized disease who had unresected or partially resected STS and were managed with radiotherapy alone for gross disease. At 5 years, local control was only 33%, with poorer overall survival [55]. An updated report from the same institution containing 112 patients (20 thigh cases) demonstrates improved local control of 45% with more modern technology [56]. In view of poor local control with conventional techniques, some centers have employed fast-neutron therapy instead of conventional photon therapy to definitively manage STS. Multicenter European data encompassing over 1,100 patients shows local control approximating 50% for patients treated definitively or adjuvantly after an R2 resection with fast-neutron therapy [57]. In the US, the University of Washington routinely employs fast neutrons for unresectable or gross residual STS and institutional analyses report 60% to 70% local control rates [58, 59]. Neither the European nor the Washington data mention results specific to the proximal lower extremity. In sum, radiotherapy alone is infrequently used for STS of the proximal lower extremity and data are scarce; however, data extrapolated from other sites suggest that local control is poor but may be improved through dose escalation or heavy-ion therapy like neutrons.

### 2.7. Reirradiation

Combined-modality treatment for STS of the proximal lower extremity yields local-control rates close to 90%; local recurrences are rare but may have a profound impact. Local recurrence is associated with poorer overall survival and distant metastasis-free survival although the mechanism of this association is unclear. Managing recurrences remains a challenge to the surgeon and radiation therapist. Recurrences can be managed by wide local excision alone or in combination with radiation delivered as external beam, brachytherapy, or both. No data specifically focusing on reirradiation of the proximal lower extremity exist, and many authors do not report tumor location or perform subgroup analyses because the number of locally recurrent patients reviewed is often quite low. 

Historically, patients undergoing reirradiation of recurrent STS have poor local control, with 5-year actuarial control rates ranging from 30% to 70% [60]. More recent data suggest that the local control for reirradiation may be worse. Six patients with proximal lower-extremity tumors have been reirradiated at the University of Florida (Gainesville) from 1965 to 2007. Of these six patients, 3 patients experienced severe complications, 2 of which required amputation. The 3 proximal lower-extremity patients who did not experience severe complications from treatment died of metastatic disease at an average of 1.5 years from retreatment. Recent data from MDACC question the role of radiotherapy in the management of recurrent disease. In a series of 62 patients with recurrent STS of various sites, local control was not significantly improved by adding radiotherapy to wide local excision. Furthermore, the addition of radiotherapy for locally recurrent STS substantially increased the rate of complications, including amputation. The data are striking; 80% of irradiated patients experienced complications necessitating medical or surgical interventions, compared to only 17% of patients managed by surgery alone [61]. Although it is difficult to extrapolate data for the proximal lower extremity, all-site STS data clearly indicate that local control is poor following a local recurrence and reirradiation must be approached with caution.

### 2.8. Complications

As with any site, multimodality treatment of proximal lower-extremity STS is prone to numerous treatment-related complications, including wound infections, persistent edema, joint stiffness, nerve damage, and femoral fracture (Table 1). As previously discussed, preoperative radiotherapy is associated with higher rates of acute wound complications, while postoperative therapy is linked to a higher risk for late complications. 

A femoral fracture is a potentially major complication following combined-modality treatment for STS of the proximal lower extremity, with incidence rates ranging from 5% to 8.6% [10, 62–64]. Other analyses including additional lower-extremity tumor sites find a lower overall incidence of fracture, but close inspection of the data reveals that thigh primaries account for most fractures [46, 62]. A femoral fracture is particularly devastating; up to two-thirds of patients never achieve bone union and those who do often require more than 1 year [65]. Numerous factors seem to be associated with fracture risk, but periosteal stripping at time of resection appears to be the strongest, with some studies reporting coincident fracture rates ranging from 20% to 30% at 5 years [10, 63, 64]. A large retrospective series from MDACC reported lower rates of fracture but did not include an analysis of periosteal stripping due to inadequacy of operative reports [46]. Contrary to evidence suggesting that periosteal stripping leads to more fractures, a large series including 239 cases of STS in the proximal lower extremity found that periosteal stripping was not necessarily associated with fracture. Rather, the analysis suggests that radiotherapy timing may play a bigger role in fracture risk, with postoperatively irradiated patients having 9 times the fracture risk [62]. Although the association is clear, this high relative rate of fracture may be a secondary effect related to the higher bone doses and larger irradiated volumes utilized in the postoperative setting [66]. Several other factors, including the use of any external-beam therapy, radiation to the entire circumference of bone, female gender, chemotherapy, marginal excision, and age greater than 50 years have also been associated with an increased risk for fracture, and the risk seems to be compounded when periosteal stripping is performed [10, 46, 62–64].

**Figure 1:**
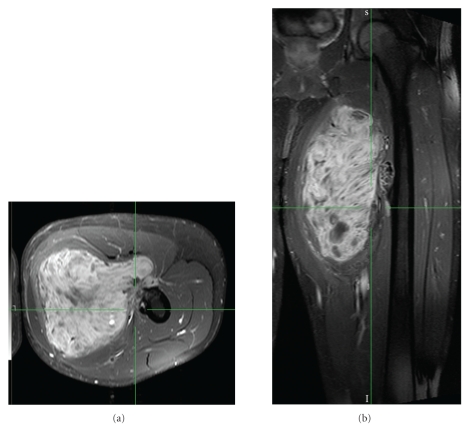
Axial (a) and coronal (b) magnetic resonance imaging scans of soft tissue sarcoma arising from the adductor muscle and invading the periosteum of the femur.

The anatomic subsite within the proximal lower extremity has also been found to correlate with risk for complications and is predictably related to critical anatomic structures located nearby (i.e., nerves, lymphatic vessels, etc.). Wound complications appear to be lower in the anterior compartment of the thigh when compared to the other compartments, while edema is more common with tumors of the medial compartment (Figure 1) [10]. Nerve damage, on the other hand, is most common in posterior-compartment tumors, likely secondary to the presence of the sciatic nerve [10]. The location of the tumor in the anterior compartment of the thigh has been associated with an increased risk of fracture—with some reports suggesting 15 times the relative risk—but this is uncertain as other investigators did not find a statistically significant increase in relative risk [10, 62–64]. The relationship between anatomic subsite and functional outcome after therapy remains unclear, with some reports suggesting greater morbidity for anterior compartment tumors [13] and others suggesting worse outcomes for tumors of the posterior compartment [9].

### 2.9. New Techniques and Therapies

Despite the numerous factors correlating with complications in multimodal management of proximal lower-extremity STS, several authors have noted that some of the more common indicators, like tumor size and anatomic location, may simply be surrogates for volume of irradiated tissue [46]. In an effort to reduce irradiated volumes of normal tissues, some radiation oncologists have begun to use IMRT techniques on the thigh with the hope of reducing complications like femoral fractures or tissue fibrosis. In a review of 10 patients with STS of the thigh, Hong et al. demonstrated that IMRT provided better conformal target coverage and decreased the exposure of surrounding normal tissues to high-dose radiation when compared to three-dimensional conformal radiotherapy (3D-CRT) plans. IMRT plans lowered the V100 of the femur by an average of 57%, which would theoretically reduce the risk for femoral fracture. Likewise, the V100 of surrounding normal soft tissues in the treated thigh were reduced by an average of 78% [67]. Despite advantages with high-dose distribution, IMRT increases the integral dose to normal tissue, particularly in the low- to intermediate-dose range. This may result in a relative increased risk of second malignancy and, if an adequate “strip” of the normal lymphatic drainage is not spared, an increased risk of lymphedema. 

Fast-neutron therapy has been used in the US and abroad, especially as previously described in the setting of definitive treatment or following incomplete resections [57–59]. Neutrons have several radiobiological characteristics that increase their utility in the treatment of sarcomas, which are frequently large rapidly growing tumors with necrotic elements. Neutrons maintain effectiveness in hypoxic environments and throughout longer periods of the cell cycle [58]. Despite the encouraging data in favor of fast-neutron therapy, the percentage of late effects remains high [59]. However, no studies have been performed looking specifically at the utility of neutron therapy in extremity sites. 

**Figure 2:**
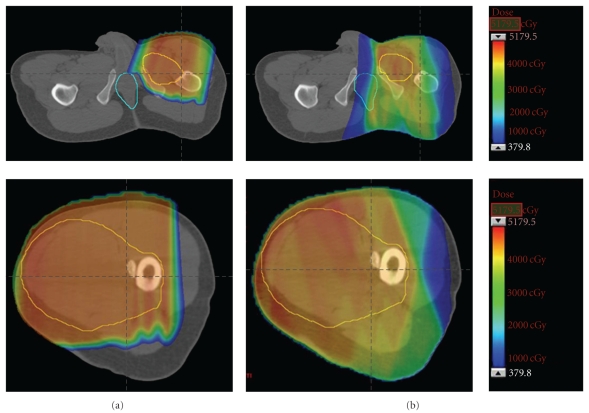
Comparison of preoperative proton (a) and photon (b) dosimetry for soft tissue sarcoma of the thigh shown in Figure 1. The tumor target volume is outlined in yellow and the anus is outlined in aqua blue. Note how the proton plan spares the perineal region and lymphatic drainage of uninvolved soft tissue.

Like IMRT, proton therapy is recognized by US cooperative groups as a viable option to improve the therapeutic ratio of radiation for STS in adults [68] and children [69]. Favorable outcomes with proton therapy have been published for sarcomas of the head and neck, skull base, and spinal cord [70]. Due to the cost and limited access to facilities, proton therapy has not been routinely used in the management of STS of the proximal lower extremity, but in select cases it may be advantageous as it offers the conformality of IMRT with the minimal integral tissue dose of 3D-CRT (Figure 2). The radiobiologic characteristics of protons are more similar to photons than neutrons and, therefore, there is a diminished concern regarding late effects.

### 2.10. Chemotherapy

In spite of the numerous studies exploring its use, the role of chemotherapy has not been firmly established for the treatment of STS. Furthermore, no data specifically focusing on outcomes for patients with primary tumors of the proximal lower extremity are available. The Sarcoma Meta-analysis Collaboration (SMAC) pooled all-site data from 14 RCTs and found improved local and distant control, but no improvement in overall survival [71]. A subgroup analysis suggested a significant overall survival benefit for patients with extremity STS; however, other investigations fail to show this correlation [72, 73]. In sum, despite the volume of data, the use of adjuvant chemotherapy in STS is debatable and guidelines suggest an individualized approach for patients at highest risk [68]. Moreover, with conflicting published data, few conclusions can be made regarding the relative effectiveness of adjuvant chemotherapy when considering the thigh.

Due in part to the conflicting data on the efficacy of adjuvant chemotherapy and the need for improved systemic disease control, some investigators have attempted aggressive neoadjuvant chemotherapy—often combined with neoadjuvant radiotherapy—to improve rates of resectability, recurrence, and survival. Although no study of neoadjuvant chemotherapy has focused on the proximal lower extremity, DeLaney and colleagues treated 48 patients with large (>8 cm) extremity tumors (70% proximal lower extremity) with neoadjuvant chemoradiation and found a survival advantage when compared to matched controls [74]. Despite the suggested survival benefit, toxicities were profound with this regimen [75]. To summarize, although no randomized trials have been performed to clarify the use of neoadjuvant chemotherapy for lower-extremity STS, guidelines suggest that neoadjuvant chemotherapy or chemoradiation are acceptable treatments when lesions are potentially resectable or when there is a concern for adverse surgical outcomes but encourage their use as part of a clinical trial [68].

## 3. Conclusion

The proximal lower extremity is the most common site for STS and surgery alone is not ideal for tumors with high-risk features. Coordinated multimodality local therapy in the form of surgery and radiation is often critical to local control, limb preservation, and functional outcome in these patients. Preoperative radiation may provide a functional benefit in long-term survivors without compromising local control. In select circumstances, adjuvant chemotherapy may augment local management. Technical advances in surgery and radiotherapy hold promise both in the primary setting and in managing the difficult scenarios of reirradiation and unresectable tumors.

## Figures and Tables

**Table 1:** Selected outcomes for proximal lower-extremity soft tissue sarcoma.

Author	Date	Number of proximal lower extremity tumors	Treatment type (%)	Median follow up (months)	Local recurrences (%)	Complications (rate)
Enneking et al. [8] University of Florida	1981	40	Surgery alone	48	6 (15%)	NR

Karakousis et al. [13] Roswell Park	1998	44	Compartment resection—29 (66%) Wide excision—15 (34%) Radiotherapy—6 (14%)	49	6 (14%)	NR

Yang et al. [4] National Cancer Institute	1998	73	Post-op RT—33 (45%) Surgery alone—40 (55%)	115/118**	6 (13%)/5 (19%)**	NR

Fabrizio et al. [25] Mayo Clinic	2000	15	Wide local excision (100%)	55	0 (0%)	NR

Vraa et al. [17] Denmark	2001	152	Amputation (18%) Wide local excision (82%) RT (21%)	52	14 (9%)	NR

O'Sullivan et al. [30] Princess Margaret Hospital	2002	98	Pre-op RT (45%) Post-op RT (55%)	39	NR	Wound complication: Pre-op—20 (45%) Post-op—15 (28%)

Virkus et al. [34] University of Florida	2002	130	Pre-op RT	71	10 (11%)*	Wound complication: 41 (26%)*

Rimner et al. [10] Memorial Sloan Kettering	2009	255	Post-op RT (BRT alone 63%, EBRT alone 31%, both modalities 6%)	71	24 (9%)	Wound reoperation—24 (9%) Edema—34 (13%) Joint stiffness—32 (13%) Nerve damage—20 (9%) Fracture—15 (6%)

*For all lower-extremity cases (both proximal and distal).

**For high/lowgrade, respectively. NR: not reported; RT: radiotherapy; EBRT: external-beam radiotherapy; BRT: Brachytherapy.
